# Primary Urothelial Carcinoma of the Prostate: A Rare Disease With Treatment Dilemma

**DOI:** 10.7759/cureus.70187

**Published:** 2024-09-25

**Authors:** Amitkumar Khwairakpam, Pratik Sharan, Santosh Yadav, Devendra Singh Mehra, Aakash Adhikari

**Affiliations:** 1 Urology, Regional Institute of Medical Sciences, Imphal, IND

**Keywords:** acute urinary retention (aur), intravesical bcg treatment, pattern of stromal invasion, primary urothelial carcinoma of prostate, radical prostatectomy

## Abstract

Primary urothelial carcinoma of the prostate (UCP) is extremely rare. The exact pathogenesis of this disease is not known. Currently, there is no consensus regarding the optimal treatment. Reporting more such cases is essential. This can help researchers better understand the disease and identify the optimal treatment. Here we report a case of primary UCP in a man who presented with acute urinary retention (AUR). We also would like to share our treatment method. A 45-year-old man presented to the emergency department with acute urinary retention. Ultrasound screening showed an echogenic soft tissue lesion in the prostatic urethra extending to the bladder neck region, with increased vascularity on color Doppler imaging. The urinary retention was relieved by Foley catheterization, The presence of growth in the prostatic urethra was confirmed by urethrocystoscopy. He underwent transurethral resection of the tumor and prostate followed by intravesical BCG (Bacillus Calmette-Guérin) instillation. We performed regular check urethrocystoscopy and there were no features of local recurrence till the last follow-up (i.e., 30 months). Even though primary UCP is rare, clinicians have to keep in mind the possibility of this disease during evaluation for lower urinary tract symptoms. Till today there is no consensus on the standard treatment protocol for UCP and the available literatures are also limited. From our case study, it appears that transurethral resection of the prostate, followed by intravesical BCG instillation, is a potential treatment modality for primary UCP, especially for patients who refused radical surgery.

## Introduction

Primary urothelial carcinoma of the prostate (UCP) is extremely rare. It accounts for only 1-4% of all prostate malignancies [1]. Most urothelial carcinomas of the prostate are secondary to bladder carcinoma. The exact pathogenesis of this disease remains unknown. It can arise from the prostatic urethra or prostatic ducts. Most patients are elderly, and the common clinical presentation is lower urinary tract symptoms, with or without gross hematuria. The staging of primary UCP is an intensely debated topic. According to Liedberg et al., it is divided into three stages: T1, tumor invasion into subepithelial connective tissue; T2, tumor infiltration into the prostate stroma, urethral corpus, or periurethral muscles; and T3, tumor invasion into corpus cavernosum, bladder neck [2]. The literature on the management of primary urothelial carcinoma is sparse. There are currently only dozens of primary cases in the literature [3]. There is no consensus regarding the optimal treatment. Treatments reported in the literature are based on experiences as single case or case series. We feel that the reporting of such rare cases is essential. This can help the researchers/clinicians better understand the disease and identify the optimal treatment. Here we report a case of primary UCP of prostate in a 45-year-old male who presented with acute urinary retention. We also would like to share the outcomes of our treatment with a more conservative approach rather than radical.

## Case presentation

**Figure 1:**
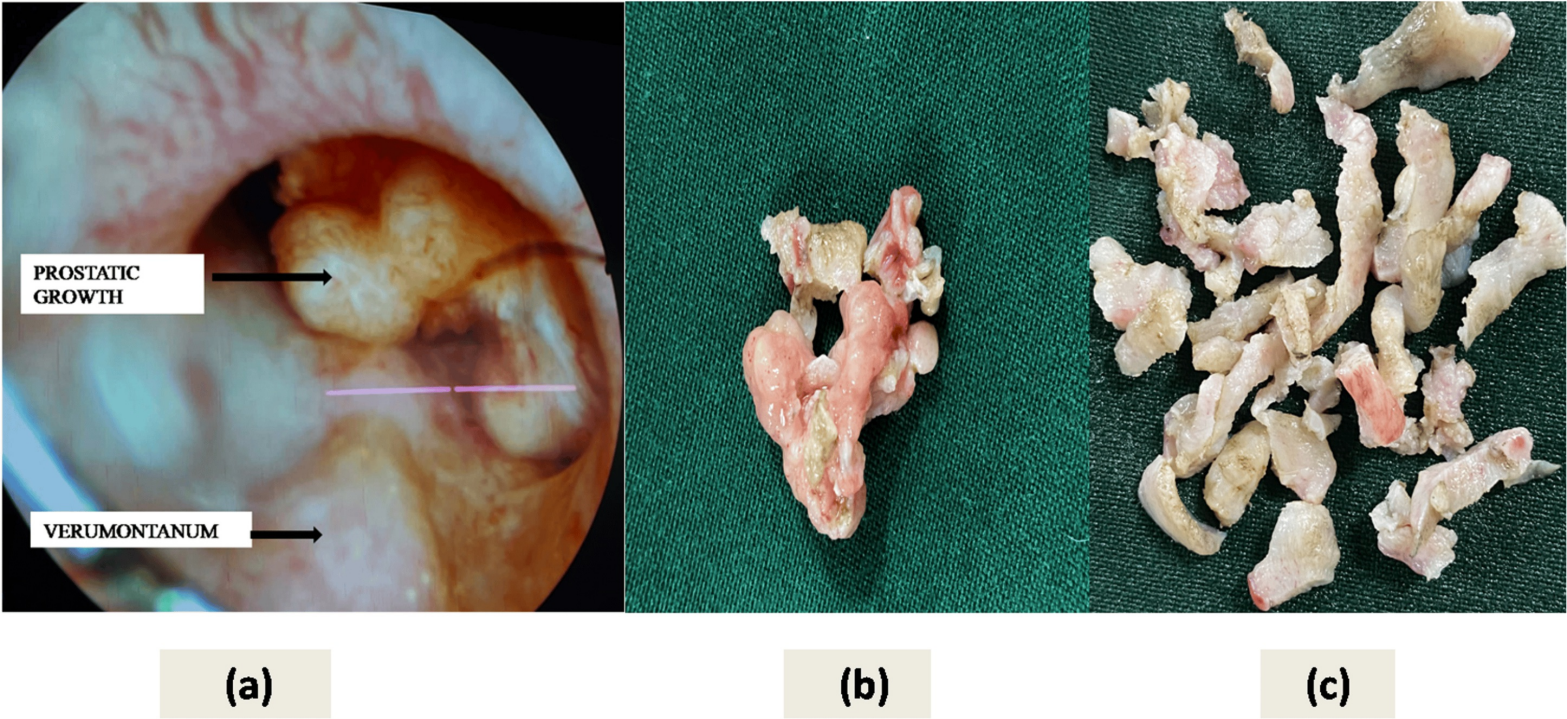
Solid-looking growth almost obliterating the prostatic urethral lumen as shown in urethrocystoscopy (a), transurethrally resected prostatic urethral growth with deep biopsy specimen (b), and resected prostate chips (c).

A 45-year-old male laborer from a remote hilly region presented to the emergency department with acute urinary retention. There was no history of lower urinary tract symptoms or episodes of gross hematuria in the past. He had been a non-alcoholic and non-smoker but a tobacco chewer for the last 15 years. On examination, there was suprapubic dullness with tenderness. On digital rectal examination (DRE) there was grade-I-prostate, non-tender, normal consistency, with no palpable nodule. Ultrasound screening revealed a hugely distended bladder with an echogenic soft tissue lesion in the prostatic urethra extending to the bladder neck region with increased vascularity on color Doppler. The urinary retention was relieved initially by putting in an 18 Fr size Foley catheter. Urethrocystoscopy under local anesthesia showed a pedunculated solid-looking growth measuring around 3 cm x 2 cm hanging from the left lateral lobe of the prostate just proximal to verumontanum almost completely blocking the prostatic urethra as shown in Figure 1a. During cystoscopy, the bladder was also found to be trabeculated but there was no growth. His serum prostate-specific antigen (PSA) was 1.5 ng/ml and the other serum biochemical parameters were within normal limits. He underwent transurethral resection of the tumor and prostate under spinal anesthesia as shown in Figure 1b and Figure 1c.

**Figure 2:**
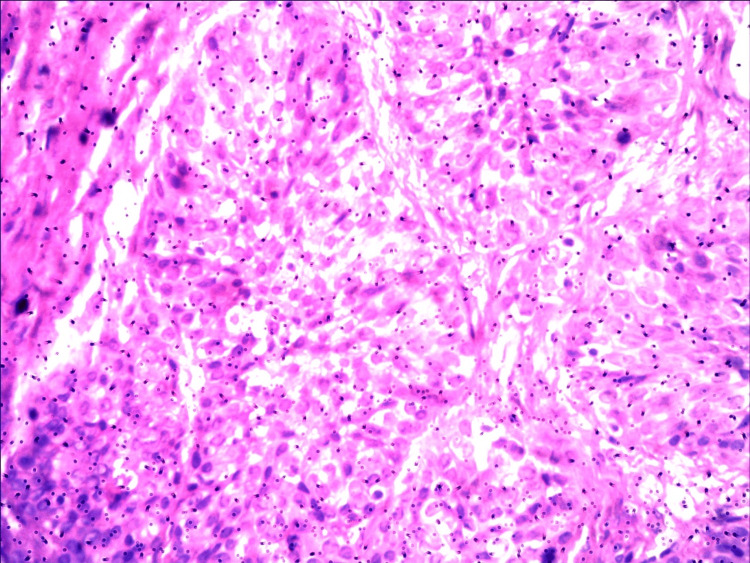
Nests and sheets of transitional tumor cells with mild to moderate pleomorphism and hyperchromatic nuclei infiltrating to muscles.

Histopathological examination (HPE) of the resected tumor revealed urothelial carcinoma, high grade with prostatic stromal involvement (Figure 2). The patient was advised for radical prostatectomy which he refused. We started him on intravesical BCG (Bacillus Calmette-Guérin) instillation (onco-BCG 40mg/ml, BCG strain, between 1-1.9 x108 Colony Forming Units) three weeks after the surgery. In the induction, 80 mg of BCG diluted in 50 ml normal saline was instilled intravesically weekly for six weeks followed by maintenance, 40 mg of BCG monthly for one year. We performed urine for malignant cytology and checked urethrocystoscopy under local anesthesia at the end of induction followed by every three months for two years and afterward every six months. There were no features of local recurrence of the tumor till 30 months of follow-up.

## Discussion

Primary urothelial carcinoma of the prostate is an aggressive neoplasm with a poor prognosis compared to adenocarcinoma of the prostate. Serum PSA levels are usually normal, and DRE findings are unremarkable in the early stage. Even magnetic resonance imaging or urethrocystoscopy may find it difficult to grasp the lesion in the early stage. Because of the above reasons, it may pose diagnostic difficulty and, hence, delayed diagnosis. More than 50% of the cases are diagnosed in advanced stages (T3, T4) [4]. In our case, the patient did not have any bothersome lower urinary tract symptoms or episodes of gross hematuria. Fortunately, during evaluation for acute urinary retention, we observed a suspicious growth in the bladder neck region on ultrasound that was confirmed on urethrocystoscopy. However, the gold standard diagnostic tool is HPE after transurethral prostate resection with biopsy with a positive rate as high as up to 90% [3]. The principles of treatment of primary UCP are extrapolated from bladder urothelial carcinoma [5]. The treatment for primary urothelial carcinoma reported in the literature varies from radical cystoprostatectomy to a more conservative surgery-transurethral resection of the prostate [6,7]. In our case, we did transurethral bipolar resection of the prostate along with the tumor till the extracapsular fat was seen followed by intravesical BCG instillation. Some scholars also believe that transurethral resection of the prostate and post-operative intravesical instillation of BCG as optimal treatments for those tumors confined to the mucosal layer or periurethral ducts, while radical prostatectomy as better treatment for primary UCP with stromal invasion [7]. Even though our patient had prostatic stromal invasion, with our treatment, he did not have features of local recurrence on check urethrocystoscopy till his last follow-up of 2.5 years. The role of chemotherapy in the neoadjuvant or adjuvant settings also remains a matter of controversy.

## Conclusions

Even though primary UCP is extremely rare, clinicians have to keep in mind the possibility of this disease while evaluating lower urinary tract symptoms or hematuria in male patients, or else the diagnosis can easily be missed. Till today, there is no consensus on its optimal treatment, and the available treatment modalities in the literature are based on case reports and case series. From our case study, it appears that transurethral resection of the prostate, followed by intravesical BCG instillation, is a potential treatment modality for primary UCP for patients who refuse radical surgery.
